# Students' Pocket and Medical Lexicon

**Published:** 1879-10

**Authors:** 


*Students' Pocket Medical Lexicon*, giving the Correct Pronunciation and Definition of all the Words and Terms in general use in Medicine and the Collateral Sciences, with an Appendix containing a List of Poisons and their Antidotes ; Abbreviations used in Prescriptions, and a Metric Scale of Doses. By Elias Longley, author of "Pronouncing Vocabulary of Geographical and Personal Names, &c." Publishers: Lindsay and Blakiston, Philadelphia, 1879.

This work is compiled in a careful and exhaustive manner, and cannot fail to afford assistance to the student in the proper pronunciation of the officinal words and terms of the profession, the study of which he is commencing, and at a time, too, when a proper knowledge of this kind is so essential. The pronunciation is plainly represented in the "American Phonetic Alphabet," preceding the text proper, together with an explanation of the alphabet. The size of this lexicon is such that it can be readily carried in the pocket, and hence can be referred to on all occasions.

